# *In Vitro* Antioxidant and Anti-Proliferation Activities of Polysaccharides from Various Extracts of Different Mushrooms

**DOI:** 10.3390/ijms13055801

**Published:** 2012-05-15

**Authors:** Xiaoyu Li, Zhenyu Wang, Lu Wang, Elfalleh Walid, Hua Zhang

**Affiliations:** 1 Harbin Institute of Technology, 73 Huanghe Road, Nangang District, Harbin 150090, China; E-Mails: smallrainlee@gmail.com (X.L.); wl1986004@126.com (L.W.); zhhua@hit.edu.cn (H.Z.); 2 Northeast Forestry University, 26 Hexing Road, Xiangfang District, Harbin 150040, China; 3 Institut des Régions Arides de Médenine, Laboratoire d’Aridoculture et Cultures Oasiennes, Médenine 4119, Tunisia; E-Mail: walid.elfalleh@fst.rnu.tn

**Keywords:** mushrooms, polysaccharides, antioxidant, anti-proliferation

## Abstract

Polysaccharides were extracted from eight kinds of Chinese mushrooms using three solvents and were evaluated for their total carbohydrate, polyphenolic and protein contents, and antioxidant and anti-proliferation activities. The results suggested that all the polysaccharides had significant antioxidant capacities (EC_50_ ranged from 1.70 ± 0.42 to 65.98 ± 1.74 μM TE/g crude polysaccharide inhibition of ABTS^+^, EC_50_ ranged from 5.06 ± 0.12 to 127.38 ± 1.58 mg VCE/g CP scavenging of OH· and EC_50_ ranged from 0.70 ± 0.04 to 33.54 ± 0.49 mg VCE/g CP inhibition of lipid peroxidation) (TE: trolox equivalent; VCE: VC equivalent; CP: crude polysaccharide). The acid extracts of *Russula vinosa Lindblad* had the highest ABTS^+^ scavenging activity. Aqueous extracts of *Dictyophora indusiata* and *Hohenbuehelia serotina* possessed, respectively, the highest OH· scavenging capacity and ability to inhibit lipid peroxidation. Mushroom extracts also inhibited proliferation of HeLa and HepG2 cells in a dose-dependent manner. These results indicate that the mushroom polysaccharides might be potential antioxidant resources.

## 1. Introduction

Historically, mushrooms were classified among the so-called lower plants in the Division *Thallophyta* by Linnaeus, largely due to their relatively simple, anatomically uncomplicated structural attributes. The early herbalists were more interested in the medicinal properties of mushrooms than in their basic value as a source of food. Mushrooms were first cited as early as in 100 B.C. in the Shen Nong’s Herbal Classic because of their medical effects. To date, mushrooms have been good source of biologically active antioxidants [1] and have been used as folk foods in China. More than 60 mushroom species have been artificially cultivated in East Asian countries. More than 30 species have been cultivated on a commercial scale in China [2]. A mushroom nutraceutical is a refined and partially defined extract from either the mycelium or the fruiting body of a mushroom, which is consumed in the form of capsules or tablets as a dietary supplement and which has potential healthcare effects [3]. Polysaccharides are one of the major compositions in the fruiting body of mushrooms and possess a lot of biological properties [4], including antioxidant, anti-tumor, anti-inflammatory [5], immunomodulation [6], the ability to lower blood glucose and cholesterol [7], *etc*. Recently, much interest has arisen in characterizing the components of water-soluble polysaccharides obtained from the fruiting bodies of mushrooms because of their ability to inhibit the growth of tumors. A major fraction of the acidic polysaccharide designated as H51 is reported to have strong antitumor activity, and structurally this component consists of a skeleton of β(1,3)-linked glucose residues, probably having branches of galactose and mannose residues and also containing acidic sugars [8].

Oxidation is essential to many living organisms for the energy production of biological processes [9]. Oxygen-derived free radicals are harmful to human health. Free radicals are highly reactive molecules with one or more unpaired electrons. During oxidative stress, free radicals are released to attack living organisms, including their DNA, RNA, protein and other substances, and eventually cause cell injury, necrosis, or apoptosis, leading to body disorder and various diseases, including cancer, cardiovascular diseases, diabetes and aging [10,11].

The aims of the present study were to determine the antioxidant and anti-proliferation activities of three solvent extracts of eight kinds of edible mushrooms commonly consumed in China. Also, several properties of the extracts, including carbohydrates, protein and total polyphenol contents were investigated. Then, the correlation of the polysaccharides contents with antioxidant activity was analyzed.

## 2. Results and Discussion

### 2.1. Total Carbohydrate, Phenolics and Protein Content of the Crude Polysaccharides

**Figure 1:**
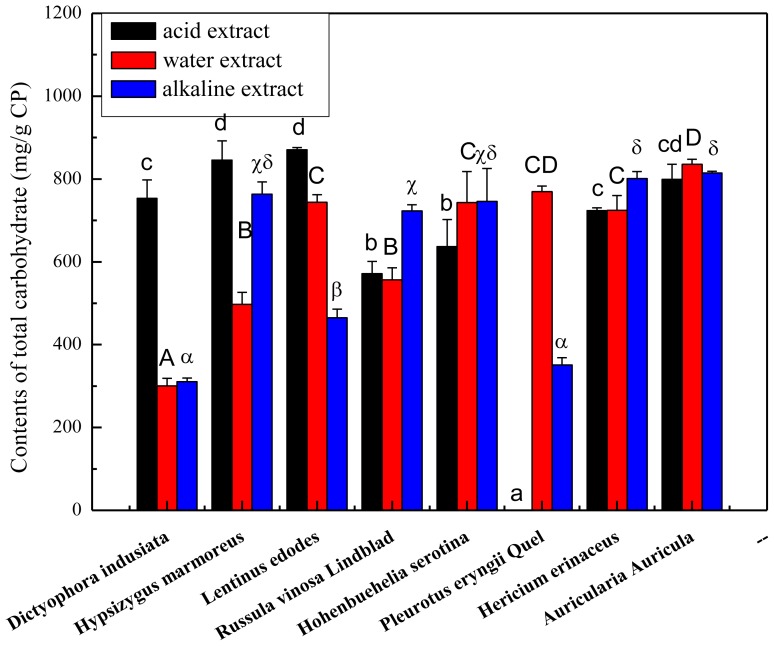
Amounts of total carbohydrates of the mushroom extracts obtained based on the phenol-sulfuric method. Total carbohydrates were expressed as mg glucose equivalent per g crude polysaccharide (means ± SD, *n* = 3). Bars with no letters in common are significantly different (*p* < 0.05).

The fruiting bodies of mushrooms were extracted with solvents of differing pH Total carbohydrate contents of the crude polysaccharides ranged from 31.05% to 87.41%, as shown in Figure 1. The highest amounts of total carbohydrates were assayed in the acid extract from *Lentinus edodes* (871.41 ± 3.54 mg/g CP), followed by the acid extract from *Auricularia Auricula* (836.05 ± 19.20 mg/g CP). Water extract of *Auricularia Aurucula* ranked third highest in terms of total carbohydrates with the total sugar exceeding 80%. The lowest amounts were observed in water extract (310.52 ± 33.04 mg/g CP) and alkaline extract (322.11 ± 15.52 mg/g CP) of *Dictyophora indusiata*. These results encourage further study to improve the extraction procedure using different solvents. Indeed, a suitable solvent extraction should be developed to recover more carbohydrates for use in the food industry.

**Figure 2:**
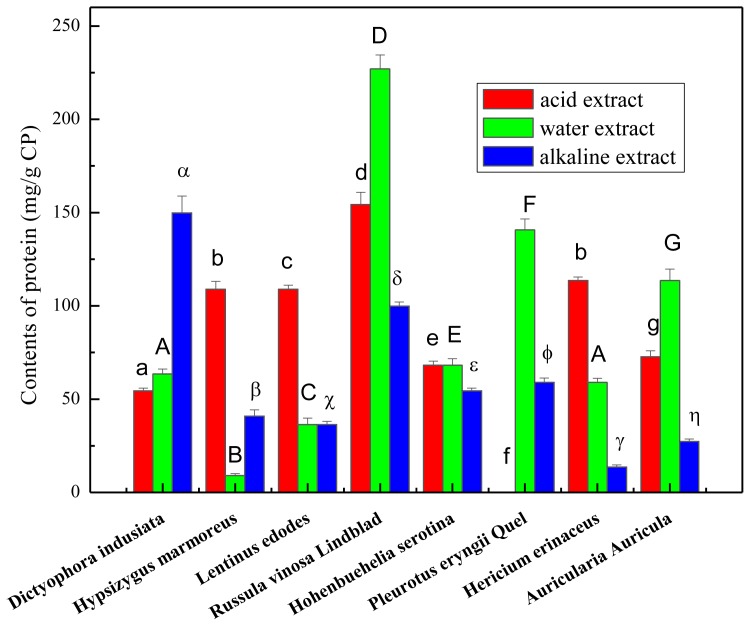
Amounts of total proteins of the mushroom extracts obtained based on the Bradford method. Total proteins were expressed as mg bovine serum albumin (BSA) equivalent per gram crude polysaccharide (means ± *SD*, *n* = 3). Bars with no letters in common are significantly different (*p* < 0.05).

Protein content of the crude polysaccharides are shown in Figure 2. Among all the samples tested, water extract from *Russula vinosa Lindblad* contained the highest level of protein (*p* < 0.05) with 227.02 ± 7.40 mg/g CP, followed by the acid extract of *Russula vinosa Lindblad* with a content of 154.37 ± 6.50 mg/g CP. Most alkaline extracts with the exception of *Dictyophora indusiata* (14.98% ± 0.9%) had protein content below 10%, with the lowest value measured for *Hericium erinaceus* (1.36% ± 0.11%; *p* < 0.05). The highest and lowest contents of protein varied about 25-fold. The results suggested that a small amount of protein was obtained from the extracts and the crude polysaccharides necessitate a deproteinization process.

Total phenols were not detected in the crude polysaccharides from mushrooms with the exception of the acid extract from *Russula vinosa Lindblad* (5.7 ± 0.2 mg GAE/g CP) and water extract from *Dictyophora indusiata* (2.9 ± 0.1 mg GAE/g CP). As expected, no phenolics were detected in most of the crude polysaccharides, suggesting that the small molecular phenolic compounds in the isolated polysaccharides had been successfully removed via precipitation and dialysis processes. This result is in agreement with Tian *et al*. [11] who reported that no phenols and flavonoids could be detected in the water-soluble polysaccharide from herbal *Houttuynia cordata.* Mattila and others [12] also proved that mushrooms contain a very low level of phenolic compounds, with flavonoid and lignan contents usually below the limits of detection.

### 2.2. Antioxidant Activity

#### 2.2.1. ABTS^+^ Radical-scavenging Activity

Polysaccharide is one of the active components in mushrooms that have multiple pharmacological activities, and one of these activities is anti-oxidation. ABTS^+^ based assay system is a rapid and efficient method for measuring the free radical scavenging activities of mushroom extracts. In this assay, the absorbance decreases as a result of a color change from blue-violet to colorless as the radical is scavenged by antioxidants [13]. The ABTS^+^ radical scavenging activities of the crude polysaccharides are presented in Table 1. It was observed that the scavenging effects of ABTS^+^ radical increase proportionally to the concentration of samples. At a concentration of 20 mg/mL, the maximum scavenging rate of these mushroom extracts ranged from 18.54% to 100%. Among all the extracts, the acid extracts of *Russula vinosa Lindblad* possessed the highest antioxidant capacities (EC_50_ 65.98 ± 1.74 μM TE/g CP; *p* < 0.05). However, the lowest antioxidant capacity was found in the acid extract of *Auricularia Auricula* (EC_50_ 1.70 ± 0.42 μM TE/g CP; *p* < 0.05). Most of the acid extracts had higher activities than the water extracts and alkaline extracts. To our knowledge, the mechanism by which polysaccharides scavenge the ABTS radical is that one molecule of ABTS radical cation extracts an electron (or hydrogen atom) from the polysaccharides and forms a semiquinone radical, regenerating the parent substrate, ABTS^+^ [14]. Subsequently, the semiquinone radical reacts with another molecule of ABTS radical cation resulting in the formation of the first polysaccharide-derived adducts. The absence of polymerization products that might be formed from coupling of carbocation radicals supports the proposal that the carbocation radicals react with the ABTS radical cation.

#### 2.2.2. OH· Scavenging Assay

Among the reactive oxygen species, the hydroxyl radical is the most reactive one and can induce severe damage to adjacent biomolecules. Hydrogen peroxide and superoxide molecules can lead to oxidative injury in the biomolecules indirectly by producing hydroxyl radicals via Fenton reaction or iron-catalyzed Haber-Weiss reaction, which can be prevented by antioxidants [15]. The abilities of the crude polysaccharides to scavenge hydroxyl radicals are shown in Table 1 and compared with that of ascorbic acid. All the OH· scavenging activities of the crude polysaccharides showed a dose-dependent pattern. Among the mushroom extracts, the water extracts of *Hypsizygus marmoreus*, *Dictyophora indusiata*, *Hohenbuehelia serotina* had the highest capacities for hydroxyl scavenging (*p* < 0.05) with EC_50_ values of 109.42 ± 1.55, 127.38 ± 1.58 and 110.64 ± 0.64 mg VCE/g CP, respectively, and the maximum scavenging rate all could reach to 100%. At the concentration of 2.0 mg/mL, the scavenging rates of the water extracts against hydroxyl radical were all higher than 50% except for the *Auricularia Auricula* extract, which was 39.49%. In contrast, the scavenging activities of all the alkaline extracts were lower than the water extracts, the alkaline extracts of *Russula vinosa Lindblad* and *Auricularia Auricula*, respectively, possessed the highest (EC_50_ 50.58 ± 0.58 mg VCE/g CP) and the lowest (EC_50_ 27.93 ± 0.55 mg VCE/g CP) OH· scavenging capacity (*p* < 0.05). Two antioxidant mechanisms of the hydroxyl radical have been implicated: one is to suppress the generation of the hydroxyl radical and the other is to directly scavenge the hydroxyl radical [16]. Thus, our results showed that the mushroom extracts, by hydrogen and/or electron donating action, might prevent reactive radical species from reaching biomolecules, such as lipoproteins, polyunsaturated fatty acids, DNA, amino acids, proteins and sugars in susceptible biological and food systems [17].

#### 2.2.3. Inhibition of Lipid Peroxidation in Rat Liver Homogenate

Lipid peroxidation is a complex process involving the interaction of oxygen-derived free radicals with polyunsaturated acids, resulting in a variety of highly reactive electrophilic aldehydes [18]. This phenomenon occurs through an ongoing free radical chain reaction until termination occurs. Free radicals attack an allylic carbon to form a carbon-centered radical. This radical reacts with O_2_ to produce peroxyl radicals (O_2_^−^). These peroxyl radicals can react with adjacent lipids forming a lipid hydroperoxide, repeating the cycle [19]. The ability of the crude polysaccharides from the mushrooms to inhibit lipid peroxidation is listed in Table 1. Results showed that administration of the crude polysaccharides decreased lipid peroxidation in liver homogenate in a concentration-dependent manner. Supplementation of water extracts from *Hohenbuehelia serotina* caused significant reduction in lipid peroxidation, which proved to have the highest activity (EC_50_ 33.54 ± 0.49 mg VCE/g CP; *p* < 0.05). The water extract of *Russula vinosa Lindblad* was found to have the lowest capacity (*p* < 0.05). Among all the extracts, water extracts of *Dictyophora indusiata* and *Hohenbuehelia serotina* and acid extracts of *Hericium erinaceus* and *Hypsizygus marmoreus* had higher inhibitory activities than the other extracts, with inhibition o more than 50%. Cheung [20] reported that the mushrooms *L. edodes* and *V. volvacea* display antioxidant behavior by scavenging peroxyl radicals, that are generated during lipid peroxidation.

### 2.3. Correlations of Polysaccharides with Antioxidant Activity

The antioxidant activities of the polysaccharides vary widely with different radical-scavenging methods, which strongly depend on the polysaccharide contents or constituents [21]. In the present research, the correlations of crude polysaccharides contents with ABTS^+^, OH· scavenging and inhibition of lipid peroxidation (LPO) capacities were analyzed, as shown in Table 2. The results suggested a positive correlation between crude polysaccharides with total antioxidant activity, and the correlation coefficient of crude polysaccharides and scavenging of ABTS^+^ (*R*^2^ = 0.401, *p* < 0.01) was higher than scavenging of OH· (*R*^2^ = 0.129, *p* > 0.05) and LPO (*R*^2^ = 0.102, *p* > 0.05). All these findings implied that extracts which had higher polysaccharide contents possessed stronger antioxidant capacities [22]. However, the monosaccharide compositions, glycosidic bond of connection and active groups were also related to the antioxidant activities of the crude polysaccharides. Wang and others reported that polysaccharides with different molecular weights and monosaccharide compositions possessed different antioxidant effects [23]. The exact mechanism underlying the antioxidant activity exerted by polysaccharides needs to be further investigated.

### 2.4. Cytotoxicity and Anti-proliferation Assay

**Figure 3:**
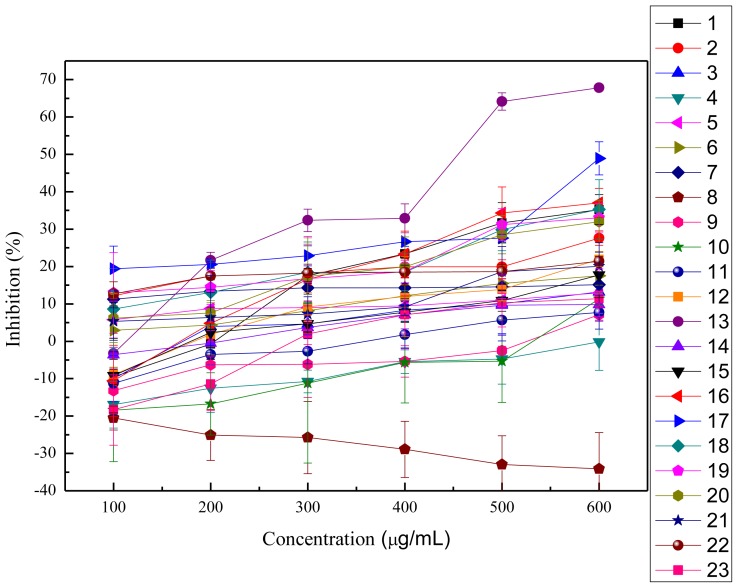
Inhibition of HeLa cell proliferation with treatment of different concentrations of mushroom polysaccharides for 48 h (means ± SD, *n* = 3). 1: acid extract of *Dictyophora indusiata*; 2: acid extract of *Russula vinosa Lindblad*; 3: acid extract of *Lentinus edodes*; 4: acid extract of *Hypsizygus marmoreus*; 5: acid extract of *Hohenbuehelia serotina*; 6: acid extract of *Hericium erinaceus*; 7: acid extract of *Auricularia Auricula*; 8: water extract of *Dictyophora indusiata*; 9: water extract of *Russula vinosa Lindblad*; 10: water extract of *Lentinus edodes*; 11: water extract of *Hypsizygus marmoreus*; 12: water extract of *Hohenbuehelia serotina*; 13: water extract of *Hericium erinaceus*; 14: water extract of *Auricularia Auricula*; 15: water extract of *Pleurotus eryngii Quel*; 16: alkaline extract of *Dictyophora indusiata*; 17: alkaline extract of *Russula vinosa Lindblad*; 18: alkaline extract of *Lentinus edodes*; 19: alkaline extract of *Hypsizygus marmoreus*; 20: alkaline extract of *Hohenbuehelia serotina*; 21: alkaline extract of *Hericium erinaceus*; 22: alkaline extract of *Auricularia Auricula*; 23: alkaline extract of *Pleurotus eryngii Quel*.

**Figure 4:**
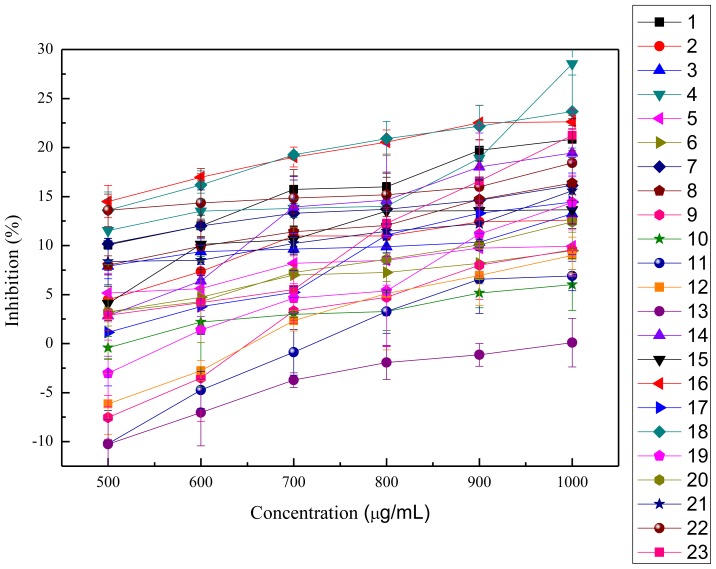
Inhibition of HepG2 cell proliferation with treatment of different concentrations of mushroom polysaccharides for 48 h (means ± SD, *n* = 3). 1: acid extract of *Dictyophora indusiata*; 2: acid extract of *Russula vinosa Lindblad*; 3: acid extract of *Lentinus edodes*; 4: acid extract of *Hypsizygus marmoreus*; 5: acid extract of *Hohenbuehelia serotina*; 6: acid extract of *Hericium erinaceus*; 7: acid extract of *Auricularia Auricula*; 8: water extract of *Dictyophora indusiata*; 9: water extract of *Russula vinosa Lindblad*; 10: water extract of *Lentinus edodes*; 11: water extract of *Hypsizygus marmoreus*; 12: water extract of *Hohenbuehelia serotina*; 13: water extract of *Hericium erinaceus*; 14: water extract of *Auricularia Auricula*; 15: water extract of *Pleurotus eryngii Quel*; 16: alkaline extract of *Dictyophora indusiata*; 17: alkaline extract of *Russula vinosa Lindblad*; 18: alkaline extract of *Lentinus edodes*; 19: alkaline extract of *Hypsizygus marmoreus*; 20: alkaline extract of *Hohenbuehelia serotina*; 21: alkaline extract of *Hericium erinaceus*; 22: alkaline extract of *Auricularia Auricula*; 23: alkaline extract of *Pleurotus eryngii Quel*.

**Figure 5:**
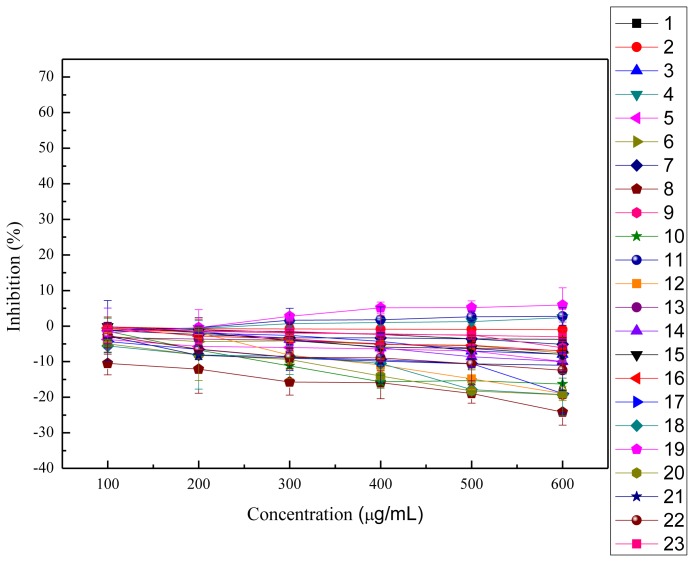
Cytotoxicity on ECV304 cell by treatment with different concentrations of mushroom polysaccharides for 48 h (means ± SD, *n* = 3). 1: acid extract of *Dictyophora indusiata*; 2: acid extract of *Russula vinosa Lindblad*; 3: acid extract of *Lentinus edodes*; 4: acid extract of *Hypsizygus marmoreus*; 5: acid extract of *Hohenbuehelia serotina*; 6: acid extract of *Hericium erinaceus*; 7: acid extract of *Auricularia Auricula*; 8: water extract of *Dictyophora indusiata*; 9: water extract of *Russula vinosa Lindblad*; 10: water extract of *Lentinus edodes*; 11: water extract of *Hypsizygus marmoreus*; 12: water extract of *Hohenbuehelia serotina*; 13: water extract of *Hericium erinaceus*; 14: water extract of *Auricularia Auricula*; 15: water extract of *Pleurotus eryngii Quel*; 16: alkaline extract of *Dictyophora indusiata*; 17: alkaline extract of *Russula vinosa Lindblad*; 18: alkaline extract of *Lentinus edodes*; 19: alkaline extract of *Hypsizygus marmoreus*; 20: alkaline extract of *Hohenbuehelia serotina*; 21: alkaline extract of *Hericium erinaceus*; 22: alkaline extract of *Auricularia Auricula*; 23: alkaline extract of *Pleurotus eryngii Quel*.

In this study, the anti-proliferation activities of the crude polysaccharides were tested on HeLa cells and HepG2 cells, as shown in Figures 3,4. The viability of HeLa cells followed a dose-dependent decline after treatment with the crude polysaccharides for 48 h. The results shown in Table 3 indicate that all the polysaccharides presented significant anti-proliferation activities against HeLa cells *in vitro* when compared to the blank control groups except for the acid extract of *Hypsizygus marmoreus*. The cell survival rates increased with increasing concentrations of acid extract of *Hypsizygus marmoreus*. This suggested that the acid extract of *Hypsizygus marmoreus* has no cytotoxicity toward HeLa cells. However, at low concentrations, most of the crude polysaccharides could promote cell proliferation, and with continuing increasing concentration, the crude polysaccharides were demonstrated to exhibit significant inhibition on HeLa cells proliferation. Among all the extracts tested, the water extract of *Hericium erinaceus* had the highest inhibitory effect, which reached 67.85%, with the EC_50_ 431.96 μg/mL. In contrast, all the extracts had low inhibition on HepG2 cells, as shown in Table 3. Based on experimental data, the acid extract of *Hypsizygus marmoreus* was found to have the highest capacity to inhibit the growth of HepG2 cells with the inhibition of 28.55%. The result indicated that the crude polysaccharides have stronger cytotoxic activity on HeLa cells than on HepG2 cells. Cytotoxicities of the polysaccharides on the normal human endothelial cell line (ECV304) were evaluated according to MTT method, as shown in Figure 5. The results suggested that the cytotoxicities of all the polysaccharides on the normal cells were much lower that that against HeLa and HepG2 cancer cells. An agent that can selectively induce cell death in tumor cells with low cytotoxicity to normal cells would be an ideal chemotherapeutic agent against cancer.

These differences in anti-proliferation activities may be attributed to their different molecular weights, charge characteristics and monosaccharide distributions [24]. Molecular weight and monosaccharide composition were considered as the two important factors related to the anti-proliferation effects of the polysaccharides. Chen and others found that polysaccharides with high molecular weight possessed stronger inhibitory effects against S180 tumor cells than polysaccharides with low molecular weight [25]. Hung and others found that polysaccharides with high antitumor activities were mostly heteropolysaccharides and had a monosaccharide composition of rhamnose, arabinose, xylose, mannose, glucose, and galactose [26]. Fujimoto and others [27] reported that the polysaccharides with excellent antitumor activities were branched glucans with (1→3)-β-, (1→4)-β-, and (1→6)-β-linkages. We could suppose that the crude polysaccharides may act as necessary nutrients to promote the development of cells at low concentrations. With increasing concentrations, the extracts may produce cytotoxic effects on cancer cells and induce apoptosis. These results indicate that bioactive compounds from mushroom extracts could be a potential source of anti-cancer drugs.

## 3. Experimental Section

### 3.1. Chemicals

All chemicals were of analytical grade and Folin-Ciocalteu (FC) reagent, 2,2′-azinobis (3-ethyl-benzothiazoline-6-sulfonic acid) diammonium salt (ABTS) were purchased from Sigma-Aldrich, Co. (St. Louis, MO). Others were purchased from local suppliers.

### 3.2. Mushrooms

Eight dried mushrooms namely, *Hericium erinaceus*, *Pleurotus eryngii Quel*, *Russula vinosa Lindblad*, *Auricularia Auricula*, *Hypsizygus marmoreus*, *Dictyophora indusiata*, *Hohenbuehelia serotina*, *Lentinus edodes* were collected from the East of China. The voucher specimens were identified by Zhenyu Wang, College of Food Science and Engineering, Harbin Institute of Technology, China.

### 3.3. Crude Polysaccharides Extraction

Fruiting bodies of eight kinds of mushrooms were smashed and passed through 200 mesh sieve. All the mushroom powders were defatted with petroleum ether overnight. The polysaccharides present in the mushrooms were extracted following the method reported by Ying [28] with a few modifications. Briefly, the ground powder of the samples was extracted in triplicate with one hundred volumes of solvents (distilled water, 1 mol/L HCl solution, 1 mol/L NaOH solution) at 80 °C with ultrasound treatment for 20 min and incubated for 3 h in a water-bath set at 80 °C. The solvent extracts obtained with the same solvent were then combined and separated by filtration on a Buchner funnel and centrifuged at 8000 *g* for 10 min. The supernatant was collected and concentrated with a rotary evaporator at 50 °C under vacuum. Then, each water extract was precipitated with three volumes of 95% ethanol overnight, and the samples extracted by 1 mol/L HCl and 1 mol/L NaOH solutions were dialyzed against running water for 1 day and distilled water for 1 day, then the extracts were re-concentrated and precipitated. All the pellets were washed with absolute ethanol, acetone and ether independently, then re-dissolved into a small amount of distilled water and lyophilized. The samples were stored at −20 °C until further analysis.

### 3.4. The Properties of Crude Polysaccharides

#### 3.4.1. Measurement of Total Carbohydrate Contents

Total carbohydrate content in crude polysaccharides was measured by phenol-sulfuric acid colorimetric method [29]. The color reaction was initiated by mixing 2 mL of crude polysaccharide solution with 1 mL of 6% phenol, followed immediately by 5 mL of concentrated sulfuric acid, and the reaction mixture was kept at room temperature for 30 min. Absorbance was read at 490 nm and total carbohydrate contents were calculated using a glucose standard calibration curve.

#### 3.4.2. Measurement of Protein Contents

The procedure used to determine the total protein content was adapted from the Bradford method [30]. An aliquot of sample (25 μL) was mixed with 1.5 mL of Bio-Rad Protein Assay kit solution, which was diluted 5 times. After shaking and incubating at room temperature for 10 min, the absorbance was read at 595 nm, and the protein content was calculated with bovine serum albumin (BSA) as standard protein.

#### 3.4.3. Determination of Total Phenolic Contents

The contents of total phenol in the crude polysaccharides were evaluated by the Folin-Ciocalteu colorimetric method [31]. Briefly, all the extracts deproteinized by Sevag method were diluted with distilled water to obtain readings within the standard curve range of 0.0 to 400 μg/mL of gallic acid. A sample (2 mL) was mixed with 1 mL of Folin-Ciocalteu reagent. Four minutes later, 1 mL of 10% (*w*/*v*) Na_2_CO_3_ was added, and the mixture was kept at 25 °C for 2 h, the absorbance of the mixture was read at 765 nm. The quantification was based on a standard curve of gallic acid and the total phenol content of the crude polysaccharides was expressed as gallic acid equivalent (mg GAE/g sample).

### 3.5. Antioxidant Activity Assay

#### 3.5.1. ABTS^+^ Radical-scavenging Activity

The abilities of crude polysaccharides to scavenge ABTS^+^ radical were tested. The analysis was performed using an ABTS decolorization assay with some modifications [32]. ABTS radical cation (ABTS^+^) was produced by reacting ABTS^+^ (7 mmol/L) with 140 mmol/L potassium persulfate solution (890 μL) and the mixture was left in the dark at room temperature for 12–16 h before use. The ABTS^+^ solution was diluted with water to an absorbance of 0.70 ± 0.02 at 734 nm. Radical scavenging activity of the tested samples was measured by mixing 0.1 mL of the different concentrations of crude polysaccharides with 1.9 mL of diluted ABTS^+^ solution, and the absorbance was measured after 6 min. The scavenging activity was calculated using the following Equation (1):

(1) $$\text{Scavenging }(\text{or inhibition})\;\text{rate }(\%)=[1-(A_{1}-A_{2})/A_{0}]\times 100$$

where *A*_0_ is the absorbance of the control, *A*_1_ is the absorbance of the samples addition and *A*_2_ is the blank absorbance without ABTS^+^ solution.

#### 3.5.2. OH· Scavenging Assay

The OH· scavenging assay was performed according to the method reported by Barahona and others [33] with slightly modifications. Hydroxyl radicals were generated from Fenton reaction of the FeSO_4_ and H_2_O_2_ system. The reaction mixture consisted of 0.25 mL FeSO_4_ (8 mM), 0.4 mL H_2_O_2_ (6 mM), 0.25 mL distilled water, 0.5 mL of various concentrations of crude polysaccharides and 0.1 mL sodium salicylate (20 mM). The mixture was incubated at 37 °C for 1 h, and then measured at 562 nm. The scavenging activity was calculated according to Equation (1); where *A*_0_ is the absorbance of the control, *A*_1_ is the absorbance of the samples addition and *A*_2_ is the absorbance of blank without sodium salicylate.

#### 3.5.3. Inhibition of Lipid Peroxidation in Rat Liver Homogenate

The inhibition effects of crude polysaccharides on lipid peroxidation were determined by the thiobarbituric acid method [34]. FeSO_4_-Vc was added to induce the liver homogenate peroxidation. In this method, 0.1 mL of crude polysaccharides (0–20 mg/mL) were mixed with 0.2 mL of 5% liver homogenate (each 20 mL homogenate solution contains 1.0 g rat liver), then 50 μL of FeSO_4_ (0.5 mM) and Vc (0.5 mM) were added. The mixture was incubated at 37 °C for 1 h. After incubation, 1.5 mL of trichloroacetic acid (15%) and thiobarbituric acid (0.6%) were added and the reaction was heated in boiling water for 15 min. The absorbance of the mixture was recorded at 532 nm. Ascorbic acid was used as the positive control. The percentages of inhibition effects were calculated according to Equation 1, where *A*_0_ is the absorbance of the control, *A*_1_ is the absorbance of the additional crude polysaccharides and *A*_2_ is the blank absorbance without liver homogenate.

### 3.6. Cytotoxicity and Anti-proliferation Assay

The antiproliferative activities of the crude polysaccharides were determined by MTT assay [35]. ECV304, HepG2 and HeLa cell lines were cultured in RPMI 1640 containing 10% fetal bovine serum (FBS), 100 IU/mL of penicillin and 100 μg/mL of streptomycin. All cell lines were incubated at 37 °C under a humidified atmosphere containing 5% carbon dioxide. Cell suspensions were seeded in 96-well plates, with each well containing 2 × 10^4^ cells. After 6 h of incubation, the medium of each well was changed to 100 μL of growth medium with various concentrations of the crude polysaccharides, and then cultivated for another 48 h; 10 μL of MTT solution were added to each well before termination at 4 h. The absorbance was read at 490 nm using an enzyme-linked immunosorbent assay plate reader. The cell proliferation of each sample was assessed for three replicas.

### 3.7. Statistical Analysis

SPSS was used for statistical evaluation. Data were expressed as means ± standard deviation. Statistical analyses were performed by one-way ANOVA. Differences at *p* < 0.05 were considered statistically significant by Duncan’s new multiple-range test.

## 4. Conclusions

On the basis of the experimental evidence, it can be concluded that all the crude polysaccharide extracts from the eight kinds of mushrooms had antioxidant activities and antiproliferative abilities against HeLa and HepG2 cells, in a dose dependent manner. However, under different conditions, antioxidants can turn into pro-oxidants, especially *in vivo*. Therefore, further work is still needed to investigate the antioxidant and antitumor efficacy of the mushroom polysaccharide *in vivo*. Moreover, the detailed mechanisms of the various health benefits of mushroom polysaccharide to humans still require intensive investigation, clinical trials based on administration of these extracts would be advisable in order to confirm our *in vitro* results, and to provide new evidence of their health benefits. Also, we do not discount the possibility that other minor compounds in the crude extracts may function as antioxidant or antitumor agents; these activities may be the result of a combined or synergistic effect of several undetermined compounds in the crude extracts. The exploration of newly cultivated mushrooms and their active ingredients with potential therapeutic value therefore remains a challenge.

## Figures and Tables

**Table 1:** The antioxidant properties of the mushroom extracts as observed by ABTS^+^ radical-scavenging capacity and OH· radical-scavenging capacity, and the inhibition of lipid peroxidation capacity. The results are expressed as means ± SD.

Mushrooms		ABTS^+^ (μM TE/g)	OH· (mg VCE/g)	LPO (mg VCE/g)
*Dictyophora indusiata*	Acid extract	24.28 ± 0.54 a	60.72 ± 0.83 a	5.61 ± 0.11 a
	Water extract	16.68 ± 0.41 ^b^	127.38 ± 1.58 ^b^	19.46 ± 0.27 ^b^
	Alkaline extract	22.35 ± 0.35 ^c^	46.01 ± 0.81 ^c^	14.28 ± 0.28 ^c^
*Hypsizygus marmoreus*	Acid extract	59.17 ± 0.18 ^d^	86.91 ± 1.02 ^d^	21.74 ± 0.78 ^d^
	Water extract	26.93 ± 0.21 ^e^	109.42 ± 1.55 ^e^	4.25 ± 0.23 ^e^
	Alkaline extract	45.98 ± 1.77 ^f,l^	46.61 ± 0.94 ^c^	1.51 ± 0.24 ^f^
*Lentinus edodes*	Acid extract	47.23 ± 0.81 ^f^	68.58 ± 1.44 ^f^	6.10 ± 0.34 ^a,g,h^
	Water extract	35.38 ± 0.52 ^g^	84.54 ± 0.27 ^g^	6.17 ± 0.16 ^g,h^
	Alkaline extract	10.97 ± 0.12 ^h^	46.48 ± 0.51 ^c^	13.98 ±0.23 ^c^
*Russula vinosa Lindblad*	Acid extract	65.98 ± 1.74 ^i^	83.93 ± 0.27 ^g^	6.62 ± 0.33 ^g^
	Water extract	9.65 ± 0.11 ^j^	96.44 ± 0.39 ^h^	0.70 ± 0.04 ^i^
	Alkaline extract	49.88 ± 2.50 ^k^	50.58 ± 0.58 ^i^	1.57 ± 0.06 ^f^
*Hohenbuehelia serotina*	Acid extract	50.01 ± 1.25 ^k^	76.41 ± 0.45 ^j^	18.58 ± 0.41 ^j^
	Water extract	45.33 ± 0.44 ^l^	110.64 ± 0.64 ^e^	33.54 ± 0.49 ^k^
	Alkaline extract	41.10 ± 1.33 ^m^	39.82 ± 0.37 ^k^	9.21 ± 0.12 ^l^
*Pleurotus eryngii Quel*	Acid extract	-	-	-
	Water extract	25.41 ± 0.35 a	100.90 ± 1.00 ^l^	10.57 ± 0.32 ^m^
	Alkaline extract	8.88 ± 0.12 ^j^	26.49 ± 0.40 ^m^	1.99 ± 0.08 ^f^
*Hericium erinaceus*	Acid extract	54.32 ± 0.35 ^n^	53.50 ± 0.61 ^n^	19.25 ± 0.34 ^b^
	Water extract	16.05 ± 0.19 ^b^	101.25 ± 1.48 ^l^	14.44 ± 0.34 ^c^
	Alkaline extract	31.66 ± 0.38 ^o^	41.26 ± 0.44 ^o^	5.97 ± 0.09 ^a,g^
*Auricularia Auricula*	Acid extract	1.70 ± 0.42 ^p^	5.06 ± 0.12 ^p^	8.70 ± 0.51 ^l^
	Water extract	14.16 ± 0.29 ^q^	59.71 ± 0.70 a	4.00 ± 0.13 ^e^
	Alkaline extract	4.00 ± 0.03 ^r^	27.93 ± 0.55 ^q^	2.63 ± 0.15 ^n^

a Value with no letters in common are significantly different (*p* < 0.05);

VCE: vitamin C equivalent; TE: Trolox equivalent.

**Table 2:** Spearmans-Rho coefficients for the correlation between the crude polysaccharides and antioxidant capacities measured by ABTS^+^, OH· and lipid peroxidation assays.

	Crude polysaccharides	ABTS^+·^	OH·	LPO
Crude polysaccharides	1	0.401 **	0.129	0.102
ABTS^+·^	0.401 **	1	0.327 **	0.346 **
OH·	0.129	0.327 **	1	0.464 **
LPO	0.102	0.346 **	0.464 **	1

a Correlation coefficient *R*^2^.

** Significantly different *p* < 0.01.

**Table 3:** Inhibition of HeLa and HepG2 cell proliferation by the treatment of crude polysaccharides for 48 h (means ± SD, *n* = 3).

Mushrooms		HeLa (% inhibition)	HepG2 (% inhibition)
*Dictyophora indusiata*	Acid extract	35.23 ± 4.00	20.83 ± 2.10
	Water extract	34.15 ± 9.70	16.37 ± 3.30
	Alkaline extract	37.03 ± 3.87	22.62 ± 0.66
*Hypsizygus marmoreus*	Acid extract	−0.15 ± 0.65	28.55 ± 4.78
	Water extract	7.74 ± 4.49	6.90 ± 1.50
	Alkaline extract	33.03 ± 3.80	14.39 ± 2.79
*Lentinus edodes*	Acid extract	13.18 ± 7.51	13.24 ± 4.16
	Water extract	11.33 ± 1.70	6.02 ± 2.64
	Alkaline extract	35.28 ± 7.95	23.68 ± 3.72
*Russula vinosa Lindblad*	Acid extract	27.59 ± 1.95	12.60 ± 2.96
	Water extract	7.13 ± 1.80	9.56 ± 2.73
	Alkaline extract	48.92 ± 4.43	14.44 ± 0.75
*Hohenbuehelia serotina*	Acid extract	12.97 ± 6.42	9.93 ± 2.97
	Water extract	21.95 ± 2.01	9.01 ± 1.86
	Alkaline extract	32.00 ± 5.62	12.41 ± 2.81
*Pleurotus eryngii Quel*	Acid extract	-	-
	Water extract	11.74 ± 6.10	13.66 ± 1.92
	Alkaline extract	11.38 ± 2.01	21.24 ± 2.62
*Hericium erinaceus*	Acid extract	17.54 ± 2.52	9.47 ± 2.97
	Water extract	67.85 ± 0.87	0.09 ± 0.47
	Alkaline extract	20.05 ± 6.30	15.59 ± 3.39
*Auricularia Auricula*	Acid extract	15.13 ± 0.45	16.14 ± 3.16
	Water extract	9.85 ± 2.46	19.45 ± 2.42
	Alkaline extract	21.44 ± 1.29	18.44 ± 3.81
